# Kanamycin Sulphate Loaded PLGA-Vitamin-E-TPGS Long Circulating Nanoparticles Using Combined Coating of PEG and Water-Soluble Chitosan

**DOI:** 10.1155/2017/1253294

**Published:** 2017-03-02

**Authors:** Sanaul Mustafa, V. Kusum Devi, Roopa S. Pai

**Affiliations:** Pharmaceutics Division, Faculty of Pharmacy, Al-Ameen College of Pharmacy, Bangalore, Karnataka 560027, India

## Abstract

Kanamycin sulphate (KS) is a* Mycobacterium tuberculosis* protein synthesis inhibitor. Due to its intense hydrophilicity, KS is cleared from the body within 8 h. KS has a very short plasma half-life (2.5 h). KS is used in high concentrations to reach the therapeutic levels in plasma, which results in serious nephrotoxicity/ototoxicity. To overcome aforementioned limitations, the current study aimed to develop KS loaded PLGA-Vitamin-E-TPGS nanoparticles (KS-PLGA-TPGS NPs), to act as an efficient carrier for controlled delivery of KS. To achieve a substantial extension in blood circulation, a combined design, affixation of polyethylene glycol (PEG) to KS-PLGA-TPGS NPs and adsorption of water-soluble chitosan (WSC) (cationic deacetylated chitin) to particle surface, was raised for surface modification of NPs. Surface modified NPs (KS-PEG-WSC NPs) were prepared to provide controlled delivery and circulate in the bloodstream for an extended period of time, thus minimizing dosing frequency. In vivo pharmacokinetics and in vivo biodistribution following intramuscular administration were investigated. NPs surface charge was close to neutral +3.61 mV and significantly affected by the WSC coating. KS-PEG-WSC NPs presented striking prolongation in blood circulation, reduced protein binding, and long drew-out the blood circulation half-life with resultant reduced kidney sequestration* vis-à-vis* KS-PLGA-TPGS NPs. The studies, therefore, indicate the successful formulation development of KS-PEG-WSC NPs with reduced frequency of dosing of KS indicating low incidence of nephrotoxicity/ototoxicity.

## 1. Introduction

KS is an aminoglycosides (AGs) second-line antimycobacterial agent [1]. KS is considerably a large molecule that is often used in injection form due to poor absorption through the gastrointestinal tract [2]. KS has a very short plasma half-life (2.5 h). In adults, KS recommended therapeutic dose is 15 mg/kg/day in equally divided intervals [3]. KS is used in high concentrations to reach the therapeutic levels in plasma and lung, which results in serious nephrotoxicity [4] and acquisition of KS resistance-TB [5], which are responsible for discontinuation of KS therapy. Due to its intense hydrophilicity, KS encounters several problems such as inadequate penetration into the cells and rapid elimination due to both efficient renal filtration and low level of association to plasma proteins [6] and belongs to Biopharmaceutics Classification System (BCS) class III. The high solubility (log⁡*P* of −3.1) makes KS a possible candidate for delivery through NPs as this would not only provide sustained release but also might demonstrate a lymphatic uptake and an averting rapid renal filtration of KS [7]. This will reduce the therapeutic dose, dose dependent side effects (such as nephrotoxicity), and acquisition of KS resistance-TB related to long term use of KS [8].

Efforts are being made to advance the current drug therapy by the use of nanoparticulate systems for controlled drug delivery. Till date, inhalable spray and freeze dry KS microparticles are reported [9]. Gold NPs are reported to confirm binding affinity and interacting region of the ssDNA aptamer for KS after intrapulmonary administration [10]. Recently, the use of polymeric NPs was encouraged to provide controlled delivery, overcome rapid elimination, and reduce toxicity over the age old delivery approaches like the tablet and transdermal patch [11, 12].

Biodegradable polymeric PLGA NPs are of particular interest from the pharmaceutical viewpoint [13, 14]. Chitosan NPs of AGs reported coupling of Chitosan NPs with dextran significantly improving encapsulation efficiency of AGs and enhancing the lung uptake of drug [15].

D-*α*-tocopheryl polyethylene glycol 1000 succinate (Vitamin-E-TPGS) is a water-soluble derivative of vitamin E, which has been found to be a good emulsifier or solubilizer or absorption enhancer of high emulsification efficiency [16, 17]. Drug-loaded PLGA-TPGS emulsified NPs have shown higher encapsulation efficiency, cellular uptake, longer half-life, and elevated therapeutic effects of the formulated drug [18].

Polymeric NPs as drug carriers are rapidly removed from the blood stream through phagocytosis by the cell of the reticuloendothelial system (RES) after their intravenous administration is one of the major problems [19, 20]. Adsorption of plasma proteins (opsonins) onto the surface of NPs is vital to the process of phagocytic recognition [19]. A commonly used method of masquerading NPs is PEGylation, the use of surface adsorbed groups which can hinder the hydrophobic and electrostatic interactions that help plasma proteins bind to NP surface and, thus, the formation of the so-called “stealth” NPs, which can escape rapid uptake by the phagocytic cells [21].

A lot of research carried out with stealth NPs focuses on intravenously administered NPs, but very modest information is known concerning the protein binding and thus in vivo pharmacokinetics and biodistribution of WSC coated NPs after intramuscular administration. Intravenous administration of liposomes and solid lipid NPs to mice caused slower renal clearance, increased half-life, and increased AUC in lung [22].

It is hypothesized that when protein binding of the NPs is decreased uptake by macrophages and tissues will be reduced [23]. Further, not much is known about the biodistribution of intramuscularly administered stealth NPs. Hydrophilic coatings (polysaccharide such as dextran) have been shown to reduce the uptake of NPs by the alveolar macrophage (AM) [15]. Particular interest has been given to chitosan due to its biocompatibility, biodegradability, and nontoxicity [24].

However, to our great knowledge, to date, this is the first comprehensive study investigating the effect of surface modified KS loaded PLGA-TPGS NPs with PEG-WSC on their pharmacokinetic, in vivo biodistribution and long circulation after intramuscular administration to rats. Thus, in the present study, we investigate the potential of KS loaded PLGA-TPGS NPs coated with PEG-WSC in augmenting sustained release of KS to help guard against acquisition KS resistance-TB and reduce toxicity.

## 2. Materials and Methods

### 2.1. Materials

KS was provided ex-gratis by Karnataka Antibiotic, Karnataka, India. Poly (lactic-co-glycolic acid) in a 50 : 50 molar ratio (MW 14,500 Da) and an inherent viscosity of 0.53 dl/g (Resomer RG 504 H) was received as gift sample from M/s Boehringer Ingelheim Pharma GmbH & Co. KG (Binger Str, Ingelheim, Germany). d-*α*-Tocopheryl polyethylene glycol 1000 succinate (Vitamin-E-TPGS) was supplied ex-gratis by M/s ISOCHEM S.A, Gennevilliers, France. Polyethylene glycol (MW 5,000 Da) and water-soluble chitosan were purchased from M/s Sigma Aldrich, Mumbai, India. The solvents such as disodium tetraborate, sodium octanesulphonate, and other chemicals employed for liquid chromatographic studies were all of HPLC grade.

### 2.2. Methods

#### 2.2.1. Determination of KS by HPLC

The chromatographic separation was performed on a Phenomenex C_18_ column (250 mm × 4.6 mm I.D., 5 mm particle size), under isocratic conditions using UV detection at 290 nm, based on the conditions described by [25] with modifications. The mobile phase was composed of 0.1 M disodium tetraborate (pH 9.0) and water (25 : 75, v/v) supplemented with 0.5 g/L sodium octanesulphonate. The flow rate was set at 1 ml/min and the injected sample volume was 20 *µ*l. The assay was linear over the KS concentration range of 120–840 *µ*g/ml. The limits of detection and quantification of KS were 60 *µ*g/mL and 120 *µ*g/mL, respectively.

#### 2.2.2. Preparation of KS Loaded PLGA-TPGS NPs

KS loaded PLGA-TPGS NPs were prepared in a ratio of (1 : 2) based on solvent emulsion-evaporation method, using Ultra Turrax IKA T25 digital high shear homogenizer [26]. In brief, 400 mg KS was dissolved in 30 ml of aqueous phase. The resulting solution was mixed with 800 mg of PLGA dissolved in 50 ml of dichloromethane. The organic solution was then emulsified with 100 ml of a TPGS (0.25%) aqueous solution, while homogenizing at 16,000 ×g for 30 min. The resulting emulsion was placed on the magnetic stirrer plate and continuously stirred at room temperature to evaporate organic solution for 8 h. The NPs were collected by centrifugation at 12,000 ×g for 30 min and washed four times with distilled water. The NPs were then lyophilized and stored at 4°C until further analysis. Blank (without drug) NPs were also prepared using the same method.

#### 2.2.3. Surface Modification of KS-PLGA-TPGS NPs

2% PEG was dissolved in the organic phase as matrix polymer and then emulsified into a TPGS 0.25% (w/v) (100 ml) solution by high speed homogenizer to form NPs suspension. In this protocol, WSC was dissolved in water to form a solution and was added to the above suspension of the NPs before freeze drying; the percentage of the WSC was calculated to make the total 0.7% (w/v) of the WSC in the final solution (100 ml) as surface modifying agent. The suspension was then lyophilized for 2 days [23].

#### 2.2.4. In Vitro Protein Binding Assays of KS-PEG-WSC NPs

The NPs protein binding was analyzed as method described previously for protein adsorption to polymeric NPs. Pooled rat plasma was taken and stored at −20°C. In brief, samples were made in different ratios of NPs suspension to plasma (60 : 40, 70 : 30, 80 : 20, and 90 : 10 (v/v)) to a total volume of 400 *μ*l. NPs suspension/plasma was incubated for 2 h at room temperature and then centrifuged at 12,000 ×g for 30 min to achieve a NPs pellet. The pellet was washed once with 500 *μ*l Mcllvaine's buffer at pH 7.5 to eliminate any extra unbound protein [27]. Then, Bradford assay was used to determine the concentration of protein that did not bind to the NPs [28].

### 2.3. Formulation Characterization

#### 2.3.1. Particle Size (*D*_nm_) and Zeta (*ζ*) Potential Measurements

The particle size of KS-PEG-WSC NPs and KS-PLGA-TPGS NPs was determined using Malvern Zetasizer Nano S90 (Malvern Instruments Ltd., U.K) and the zeta potential was measured using Laser Doppler Velocimetry (LDV) with a Malvern Zetasizer Nano ZS (Malvern Instruments Ltd., U.K). Samples were diluted in MilliQ™ water before measurement.

#### 2.3.2. Transmission Electron Microscopy

The morphology of the KS-PEG-WSC NPs was observed using transmission electron microscope (TEM) attached with a mega view II digital camera (H 7500, Hitachi, Tokyo, Japan). A drop of sample diluted with water was placed on a copper grid and the excess was drawn off with a filter paper. Samples were subsequently stained with 2% of uranyl acetate solution for 30 s. The image was magnified and focused on a layer of photographic film.

#### 2.3.3. Drug Encapsulation Efficiency (*D*_EE_)

The *D*_EE_ was determined by the separation of KS-PEG-WSC NPs and KS-PLGA-TPGS NPs from the aqueous medium containing nonassociated KS by ultracentrifugation (REMI high speed, cooling centrifuge, REMI Corporation, India) at 15,000 ×g for 30 min, at 4°C. The unencapsulated KS was determined using HPLC.

#### 2.3.4. In Vitro Drug Release Studies

Release studies were carried out for KS-PEG-WSC NPs and KS-PLGA-TPGS NPs. Drug-loaded NPs corresponding to 500 mg of drug were placed in dialysis cellulose membrane bags (cellophane membrane, molecular weight cut-off 10,000–12,000, Hi-Media, India). The bags were sealed closely by clamps after 1 ml of Phosphate Buffer Solution (PBS) was dropped into each bag. The dissolution medium consisted of a PBS (0.1 M, pH 7.4). The stirring rate was kept constant at 50 rpm, as was the temperature at 37°C with continuous magnetic stirring. At selected time intervals, aliquots were withdrawn from the release medium and replaced with the same amount of fresh PBS and concentrations of the released drug were determined by HPLC method.

#### 2.3.5. Pharmacokinetic Studies

All the animal investigations were performed as per the requisite protocol approved by the Institutional Animal Ethics Committee [Letter no AACP/IAEC/Jun-2014-01]. The Committee is duly approved for the purpose of control and supervision of experiments on the animals by the Government of India. The pharmacokinetic study involved two groups. Six rats (male Wistar, weighing 250–300 g) were randomly distributed amongst each group.

Group I received KS-PLGA-TPGS NPs formulation redispersed in 1 ml of water.

Group II received KS-PEG-WSC NPs formulation redispersed in 1 ml of water.

All the animal groups received a dose equivalent to 15 mg of KS per kg of body weight [3]. After intramuscular drug administration, the rats kept in cages were allowed access to food and water ad libitum. Serial aliquots of the blood samples (100 *μ*l each) were withdrawn from the retroorbital plexus under mild ether anesthesia at 0, 0.5, 1, 3, 6, 12, and every 24 h for 6 days in the heparinized microcentrifuge tubes (50 units heparin/ml of blood). Plasma was harvested by centrifugation at 15,000 ×g for 15 min and stored at −20°C until analyzed. Acetonitrile was added to precipitate the plasma proteins. Thereafter, samples were vortexed and centrifuged at 15,000 ×g for 20 min and were analyzed by HPLC. The competence of nanoparticulate formulation was assessed by administering pure drug intramuscularly and measuring the blood levels at 0, 0.5, 1, 2, 3, 6, 12, and 24 h. Noncompartmental pharmacokinetic parameters for extravascular input, that is, *C*_max_, *T*_max_, AUC_0–∞_, *T*_1/2_, *K*_*e*_, and MRT, were computed by choosing Kinetica 5.0.11 version software (Thermo Fisher Scientific Inc. Waltham, USA).

#### 2.3.6. In Vivo Biodistribution Assay

The animals and dosing protocol were the same as in pharmacokinetic study. To study in vivo biodistribution, 18 rats were randomly divided into two groups of 9 rats. Group I was treated with KS-PLGA-TPGS NPs and Group II was treated with KS-PEG-WSC NPs. Intramuscular administration was performed on the same day and the three rats of each group were sacrificed by cervical dislocation under general pentobarbital anesthesia on Day 1, Day 5, and Day 7. Heart, lungs, kidneys, and spleen as well as plasma were collected and processed immediately for analysis by HPLC [22, 25].

#### 2.3.7. Statistical Analysis

One-way ANOVA was used to compare the data. The differences in the means were significant and post hoc pair wise comparisons were conducted using Newman-Keuls multiple comparison using GraphPad Prism software ver 5.0 (M/s GraphPad Software Inc., California, USA). For in vivo biodistribution analysis was confirmed by Bonferroni's as a post hoc test and the one-way ANOVA was used to compare the data using GraphPad Prism software ver 5.0 (M/s GraphPad Software Inc., California, USA) [26].

#### 2.3.8. Stability Studies

KS-PLGA-TPGS NPs and KS-PEG-WSC NPs were subjected to stability studies, carried out at 25 ± 2°C/60%  ±  5% RH, as per the ICH guidelines for the climatic zone IV. The formulation was assayed periodically, at the time points of 0, 1, 3, and 6 months, for particle size, drug encapsulation efficiency, and zeta potential [26].

## 3. Results

### 3.1. Particle Size and Drug Encapsulation Efficiency

The solvent emulsion-evaporation method was successfully employed to fabricate NPs for intramuscular drug delivery. The yield of the polymeric NPs was 94.37% with this protocol. The KS-PLGA-TPGS NPs formed were uniform and discrete and of average particle size of ~86.71 nm with a polydispersity index less than 0.267. Postinclusion of PEG-WSC made only a 25 nm raise of the particle size (113.16 nm). KS encapsulation efficiency in PLGA-TPGS NPs and PEG-WSC NPs was found to be 66.31 ± 4.12% and 74.45 ± 2.83%, respectively. The presence of PEG-WSC modifier significantly improved the drug encapsulation efficiency; the reason is that the drug was entrapped within the PEG and WSC chains or this may be the effect of emulsification outcome of the PLGA-TPGS [13].

### 3.2. Transmission Electron Microscopy

**Figure 1:**
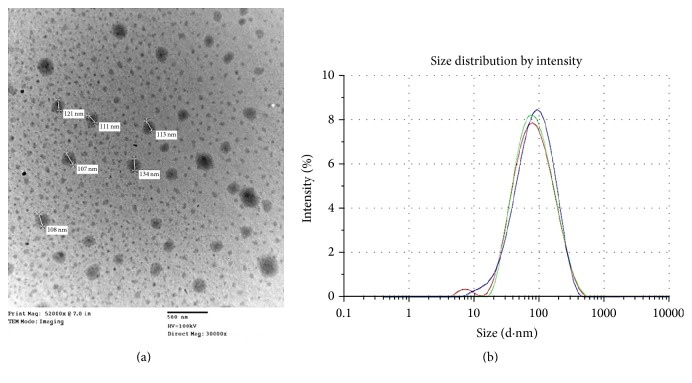
(a) TEM image shows particle size of KS-PEG-WSC NPs formulation. (b) Size distribution of KS-PEG-WSC NPs formulation. KS-PEG-WSC NPs: Kanamycin-polyethylene glycol-water-soluble chitosan-nanoparticles; TEM: transmission electron microscopy.

The TEM image (Figure 1(a)) clearly reveals that most of the emulsion particles of KS-PEG-WSC NPs formulation were below 200 nm in size and were spherical in shape.  Figure 1(b) illustrates the Dnm distribution and markedly reveals the globule size of the OPT formulation as 113.16 nm.

### 3.3. In Vitro Protein Binding of KS-PLGA-TPGS NPs and KS-PEG-WSC NPs

Different ratios of NPs: plasma were taken to evaluate the Vroman effect. This relates to a possible adsorption of proteins on the surface sites of NPs [29]. At a 60 : 40, 70 : 30, 80 : 20, and 90 : 10 plasma volume, KS-PLGA-TPGS NPs showed an average protein binding of 19.72 ± 6.19%, 27.62 ± 3.45%, 31.64 ± 4.81, and 39.12 ± 4.24%, respectively. A similarity between this formulation and a similar formulation coated with PEG- (2%) WSC (0.7%) illustrated highly momentous variation in plasma protein binding 16.41 ± 3.45 (^*∗∗∗*^*p* < 0.001) at a plasma volume of 40%. The improved protein binding for KS-PEG-WSC NPs noticed at 30% (18.73 ± 3.26%), 20% (21.74 ± 4.21%), and 10% (29.44 ± 2.76%) plasma volume was an unpredicted result since surface modification with WSC is well documented to decrease protein adsorption [30]. Statistically, no significant difference was observed between the three percentages (30%, 20%, and 10%) (^*∗*^*p* > 0.01).

### 3.4. In Vitro Drug Release Studies

**Figure 2:**
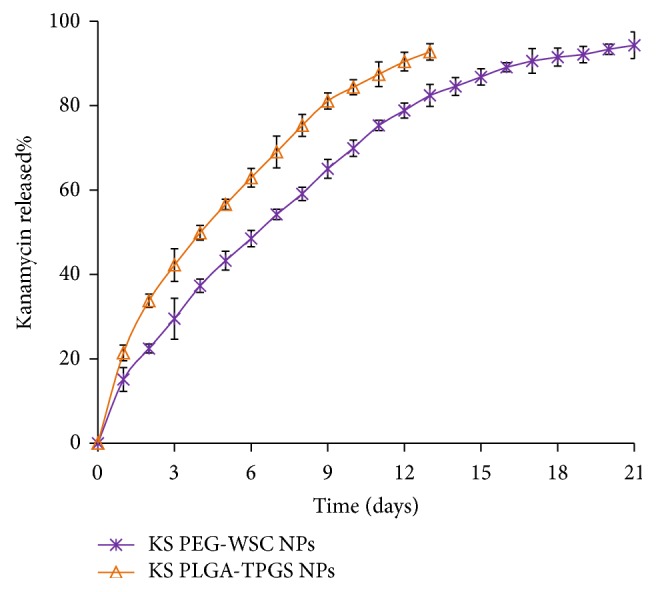
In vitro release profile of KS-PEG-WSC NPs and KS-PLGA-TPGS NPs in pH 7.4 PBS. Data represented the mean ± SD, *n* = 3.

Figure 2 presents the release profiles of KS from the PLGA-TPGS NPs and PEG-WSC NPs. KS-PLGA-TPGS NPs tended to release minuscule amount of drug ~21.42% within initial 24 h, with relatively 91.56% of drug being released within 14 days, whereas KS-PEG-WSC NPs showed ~15.11% amount of drug release within initial 24 h, followed by a slow and sustained release (93.26%) till 21 days.

### 3.5. In Vivo Pharmacokinetic Assay

**Figure 3:**
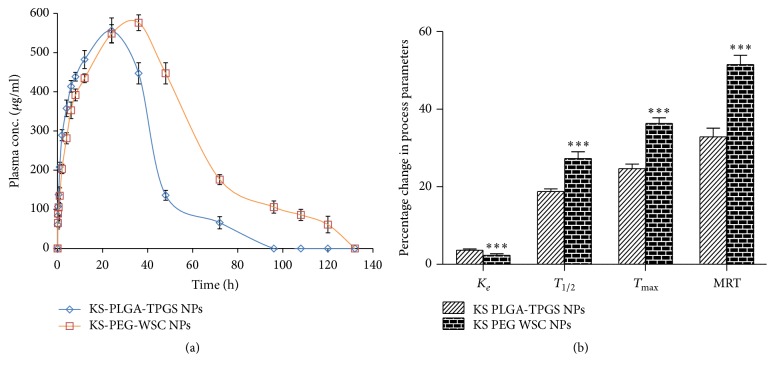
(a) Plasma drug level profiles of KS-PEG-WSC NPs and KS-PLGA-TPGS NPs. Each point represents mean of six replicates and each crossbar indicates 1 SEM and (b) change in pharmacokinetic parameters of KS-PEG-WSC NPs relative to KS-PLGA-TPGS NPs.

KS-PEG-WSC NPs showed prolonged residence in the blood circulation* vis-à-vis* KS-PLGA-TPGS NPs. The mean plasma concentration-time profiles (Figure 3) portray considerable higher plasma levels from KS-PEG-WSC NPs (^*∗∗∗*^*p* < 0.001) formulation with respect to KS-PLGA-TPGS NPs. KS-PEG-WSC NPs remained in the blood for an extended period of time (half-life,* t*_1/2_: 27.76 h) vis-à-vis KS-PLGA-TPGS NPs (half-life,* t*_1/2_: 18.23 h). The absorption rate of KS-PEG-WSC NPs was markedly enhanced, as observed by KS discrete improvement in various pharmacokinetic parameters, that is, *T*_max_, *T*_1/2_, *C*_max_, and AUC_0–∞_. Subsequent one-way ANOVA carried out on various pharmacokinetic parameters, namely, *T*_1/2_,* K*_*e*_, AUC_0–∞_, and *T*_max_, implied highly statistically significant variation (^*∗∗∗*^*p* < 0.001) in the drug absorption potential of KS from PEG-WSC NPs. As is evident from Figures 3(a) and 3(b), the availability of KS in case of PEG-WSC NPs was almost 1.52-fold as compared to PLGA-TPGS NPs up to 6 days, suggesting a longer circulation time of KS in the blood.

### 3.6. In Vivo Biodistribution Assay

**Figure 4:**
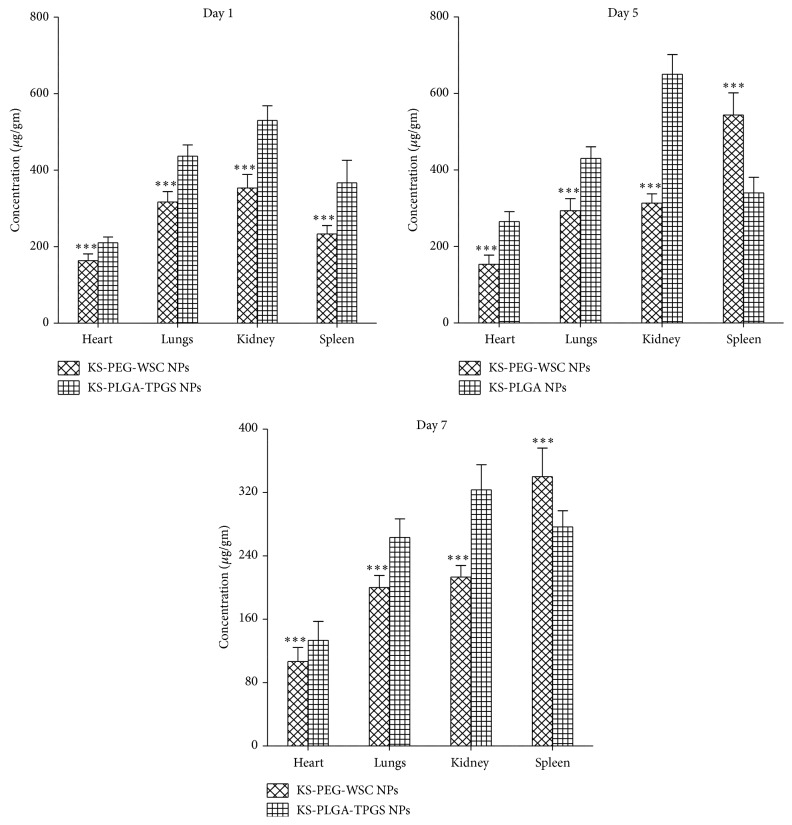
Body distribution of KS-PEG-WSC NPs and KS-PLGA-TPGS NPs after intramuscular administration to rats. Concentration of KS (*y*-axis) was determined in different organs on Day 1, Day 5, and Day 7 after intramuscular administration.

The in vivo biodistribution of the NPs was greatly influenced by PEG-WSC content. KS-PLGA-TPGS NPs without coating were detected in lungs, kidneys, heart, and the spleen over a period of 7 days (Figure 4). However, at the end of Day 1, faster elimination of marketed product was observed in all the organs* vis-à-vis* KS-PLGA-TPGS NPs and KS-PEG-WSC NPs. Minuscule amount of concentrations was detected in the plasma over the same period. It was observed that lung accumulated major portion of the administered KS-PLGA-TPGS NPs but lower lung concentration in comparison to KS-PEG-WSC NPs. The accumulation and clearance of the NPs in different organs varied. Besides the blood, the KS-PLGA-TPGS NPs were mainly found in all the heart, kidney, and spleen.

The cumulative amounts of KS PEG-WSC NPs dramatically reduced in kidney, changed little in heart and lung, and were exhibited slightly higher in spleen. The accumulation rate of KS-PEG-WSC NPs in kidney was 3.64-fold lower than that of KS-PLGA-TPGS NPs over a period of 7 days (Figure 4). The minuscule amount of concentrations in organs especially kidney indicated slower filtration of KS-PEG-WSC NPs by and extended residence time in the blood.

### 3.7. Stability Studies

KS-PLGA-TPGS NPs and KS-PEG-WSC NPs show very small variation in the formulation parameters during 6 months of storage period (Table 1).

## 4. Discussion

KS loaded PLGA-TPGS NPs surface modified with PEG and WSC was prepared in our laboratory to achieve a prolongation in systemic circulation and increased uptake in lungs (site of action). Coating was applied to prepare long circulating NPs with biodegradable polymer PLGA working as the core for drug reservoir and the water-soluble PEG and WSC accommodative as the corona designing towards the outer aqueous environment. PEG has been widely employed in drug delivery to augment the circulation time of NPs in blood. Perhaps due to electrostatic interactions between the positively charged WSC and the negatively charged PLGA, WSC coating was formed of the NPs. In addition, due to its amphiphilic nature, WSC was prone to form monolayer arrangement at the surface of NPs [31].

To our best knowledge, there has been hardly ever report on the steadiness of attaching WSC on the surface of KS loaded PLGA-TPGS NPs. A potential explanation for the positive role of the WSC coating at improving particle stability was based on an efficient steric repulsion effect produced by the hydrophilic chains of the macromolecular WSC projecting towards the external phase, which inhibited coalescence or flocculation.

This study however focused on the effect of PEG and WSC on the in vitro protein binding as well as the pharmacokinetics and in vivo biodistribution of KS-PLGA-TPGS NPs after intramuscular administration. In the present study, the augment in particle size, improvement of surface hydrophilicity, and reversal of surface charge were attained by successful “insertion” of PEG or the outer WSC layer of the NPs.

Increase in the particle size of the NPs was observed by using PEG (5,000 Da, 2%) and WSC (0.7%) in KS-PLGA-TPGS NPs, which might be owing to the multilayer depositions of PEG-WSC. This enhance in particle size was a first sign of the attachment of WSC to the surface of the NPs. The uptake of the NPs through AM decreases with increasing the particle size reaching a cut-off at around 200 nm [32]. The inclusion of positively charged WSC has been suggested in earlier reports to enhance uptake through AM [33, 34]. It has also been reported that particle size less than 200 nm generally remains undetectable to the reticuloendothelial system (RES) and keeps on circulating over an extended period of time [35].

KS-PLGA-TPGS NPs had a negative charge of −27.91 mV, which was reversed to positive value (+3.61 mV) after insertion of PEG-WSC. Zeta potential for the KS-PEG-WSC NPs was lower than the KS-PLGA-TPGS NPs (^*∗∗*^*p* < 0.001); it may be because of a higher concentration of PEG and inclusion of positively charged WSC adsorbed on the surface of NPs with the development of a denser surfactant coating on the NPs surface, thus, eliciting a reduced electrophoretic mobility, and an elevated absolute potential value ensures a high-energy barrier that stabilizes the nanosuspension [36]. NPs charge also influences their circulation time and clearance. However, the neutral NPs are anticipated to remain in circulation over a prolonged period of time as opsonization processes are not uniformly activated (as for charged NPs) for their elimination from the body [35].

PLGA-Vitamin-E-TPGS NPs have been reported to give high encapsulation efficiency for water-soluble drugs. Present investigation also reveals that solvent emulsion-evaporation method can lead to NPs with high encapsulation with water-soluble drug such as KS. The findings are in good agreement with literature reports [37].

In vitro protein binding assay was assessed to predict the phagocytosis of NPs by the cells of MPS. Phagocytic uptake of NPs by cells of MPS is responsible for the loss of NPs in the systemic circulation. A relationship between surface charge and opsonization was showed in vitro that neutrally charged NPs have a much lesser opsonization rate than charged NPs. Their lack of binding with opsonins would be responsible for their prolonged residence in the blood circulation. On the contrary, KS-PLGA-TPGS NPs with a higher negative zeta potential would possess a stronger affinity for plasma proteins, accounting for its relatively higher protein binding. It is indicated that when PLGA-TPGS NPs made long circulating, the protein binding differs depending on the polymeric surfactant used. The diminution of protein binding might be ascribed to the higher surface coverage which is achieved as a result of the conformation of the PEG-WSC chains in a “brush-like” configuration [38]. Thus, the PEG in combination with WSC was supposed to assume a dense brush-like conformation and act to increase the opsonins resistant properties. Their lack of binding with opsonins and then escaping phagocytosis would be likely responsible for their prolonged persistence in the blood circulation.

A sustained release pattern is a key issue in the development of colloidal drug delivery systems used in the field of nanomedicine. The in vitro release behavior of the KS-PLGA-TPGS NPs and KS-PEG-WSC NPs is shown in Figure 2. The initial burst release was prominent for KS-PLGA-TPGS NPs and KS-PEG-WSC NPs during the first day of release, being greater than 10–15%. This effect can be because of the drug adsorbed on the surface of NPs [39]. Besides, we believe that burst release may be accounted for the passage of very small NPs which may have passed liberally across the dialysis membrane (~10–20 nm). The initial phase of drug release however can be used fruitfully to provide a loading dose wherein ~25% of the total KS present in the formulation is released within 24 h of its administration, while the remaining amount may help to maintain the plasma concentrations for >24 h. Release of the encapsulated KS from PLGA matrix was found to occur through diffusion-cum degradation-mediated process [40]. During the early phases, release occurs mostly through diffusion in the polymer matrix, while during the later phases release is mediated through both diffusion of the KS and degradation of the polymer matrix itself. It is apparent that both the emulsifier and the particle material composition determine the in vitro release behavior. Faster drug release of KS-PLGA-TPGS NPs may be attributed to the lower molecular weight of PLGA and the higher hydrophilicity of TPGS copolymer in comparison with the KS-PEG-WSC NPs.

In the current work, KS-PEG-WSC NPs were prepared and administered intramuscularly to Wistar rats that contained ~16.21% free KS (74.51 ± 1.84%, encapsulation efficiency) and significant percentage of particles were of a very small size (113.16 nm) such that they can be transported via lymphatic transport. The neutrally charged KS-PEG-WSC NPs do not activate any system for their elimination from the body such that they are projected to bypass AM pickup resulting in extended circulation times and since lung is the major site of action of KS, AM avoidance of KS-PEG-WSC NPs is especially expected to reduce its elimination from the body. High plasma concentration complemented with overcoming of AM uptake will present higher amounts of KS in the systemic circulation. The higher *T*_max_ levels coupled with higher values of *T*_1/2_ and AUC_0–∞_ observed in those KS-PEG-WSC NPs indisputably vouch distinct improvement in rate and extent of drug bioavailability. This augmentation in bioavailability would finally result in an increase in the intensity of therapeutic effect of KS.

A significantly (^*∗∗∗*^*p* ≤ 0.001) lower concentration of KS in kidney when administered in the form of NPs indicates a lower elimination of KS. This coupled with the bypass of rapid elimination of KS due to its entrapment within PEG-WSC NPs can suitably explain a significant decrease in* K*_*e*_ of KS (^*∗∗∗*^*p* ≤ 0.001) when administered as KS NPs. This effect may be attributed to the use of larger molecular weight PEG polymer that led to longer blood circulation half-lives for the particles in vivo [18].

The in vivo biodistribution of the NPs influenced by surface PEG-WSC content in different organs varied. The concentrations of KS-PEG-WSC NPs compared with KS-PLGA-TPGS NPs significantly reduced in kidney and other organs. The major pathway for the exclusion of NPs from blood appeared to be the NPs detention in MPS organs. Typically, once polymeric NPs are opsonized and separated from the bloodstream, they are sequestered in one of the MPS organs. In the case of NPs lack of stealth properties, accumulation in the MPS organs is very fast within a minute and generally concentrates in the kidney and spleen [16].

The significant reduction in kidney for KS-PEG-WSC NPs may be due to the hidden surface created by PEG-WSC coating, with neutral-charge and brush-like conformation, which could decrease kidney filtration. A small high spleen uptake was considered to be a result of prolonged systemic circulation of KS-PEG-WSC NPs [30]. Though, we agree that PLGA-TPGS NPs do show a significant prolonged circulation, nevertheless the effect was much more prominent in case of a PEG-WSC coating at all time point studies. Thus, an advantage of PEG-WSC coating on the NPs for circumventing AM uptake was obvious. A relatively high accumulation of NPs in spleen was observed compared with KS-PLGA-TPGS NPs and extended residence time in the blood might indicate slower kidney filtration.

NPs were detected in heart, lung, kidney, and spleen over the 5 days and the plasma concentrations of KS-PEG-WSC NPs were higher than that of KS-PLGA-TPGS NPs, demonstrating that the extended residence time can be obtained with intramuscular formulations. This effect may be due to surface heterogeneity and hydrophobic nature of PLGA which could further explain the presence of NPs in the spleen (representing particles that are taken up by the M cells of the Peyer's patches via opsonization) and the kidney (representing particles size > 10 nm averting rapid kidney filtration).

The KS-PEG-WSC NPs resulted in reduced protein binding when administered intramuscularly and there is a significant increase in the percentage detected in plasma; the distribution to various organs is statistically significantly (^*∗∗∗*^*p* < 0.001) different from the KS-PLGA-TPGS NPs. Furthermore, these results designate that for NPs in vitro observations cannot represent or be correlated to the in vivo behavior of the NPs.

## 5. Conclusions

In the present study, PLGA-TPGS NPs bearing a combined coating of PEG and WSC were prepared. Surface charge and surface hydrophilicity were significantly affected by the nature of the coating. Combination of PEG and WSC presented an effective shielding character to the NPs as well as neutral surface charge. Also, entrapping KS into PEG-WSC NPs could result in improved availability of KS in lung (sites of action), or minimum concentration in kidney (sites of rapid clearance as well as toxicity). Based on the above results, these NPs might be adequate for good long circulating efficacy and will be promising scenario as efficiently targeted drug delivery systems.

## Figures and Tables

**Table 1:** Various parameters of the KS-PLGA-TPGS NPs and KS-PEG-WSC NPs analyzed at different time points during stability studies.

Time (month)	*D*_nm_		*D*_EE_ (%)		*ζ* potential	
	KS-PLGA-TPGS NPs	KS-PEG-WSC NPs	KS-PLGA-TPGS NPs	KS-PEG-WSC NPs	KS-PLGA-TPGS NPs	KS-PEG-WSC NPs
0	86.71	113.16	66.31	74.45	−27.91	+3.61
1	86.19	113.03	65.85	74.21	−27.67	+3.53
3	85.72	112.86	65.51	73.83	−27.32	+3.02
6	85.36	112.45	65.22	73.26	−27.13	+2.98
